# Nucleation and growth of primary nanostructures in SrTiO_3_ homoepitaxy

**DOI:** 10.1186/s11671-015-0805-7

**Published:** 2015-02-26

**Authors:** Soo-hyon Phark, Young Jun Chang

**Affiliations:** Center for Correlated Electron Systems, Institute for Basic Science (IBS), Seoul National University, Seoul, 151-747 Republic of Korea; Department of Physics and Astronomy, Seoul National University, Seoul, 151-747 Republic of Korea; Department of Physics, University of Seoul, Seoul, 130-743 Republic of Korea

**Keywords:** Island nucleation, Scanning tunneling microscopy, Perovskite homoepitaxy, Surface reconstruction

## Abstract

SrTiO_3_ nanoislands on SrTiO_3_ (001) in a *diffusion-limited growth* regime were studied using *in situ* scanning tunneling microscopy (STM). The STM images revealed two characteristic features of nucleation stages. First, the minimum lateral size of the one-unit-cell (*uc*)-high SrTiO_3_ islands was 4 × 4 *uc*^2^. Second, one-dimensional SrTiO_3_ islands of a 4 *uc* width grew along the crystal symmetry directions. These observations suggest that 4 × 4-*uc*^2^ islands act as a minimum nucleation seed, and the addition of SrTiO_3_ molecular species of the same width is the primary and dominant growth process in SrTiO_3_ homoepitaxy. A close inspection of the surface of the substrate during the deposition process revealed possible connections between surface reconstruction and energetically favorable nucleation of SrTiO_3_ islands.

## Background

Atomic-scale control over oxide surfaces and interfaces is one of the most important technological advances required to exploit the functionality of oxide materials. SrTiO_3_ is one of the most widely studied oxide materials and has been the focus of research interest in the metal-insulator transition, [1] ferroelectricity [2,3], and superconductivity [4]. Recent advances in oxide film deposition techniques, including pulsed laser deposition (PLD) and molecular beam epitaxy, have enabled the formation of atomically flat surfaces, as well as controlled film-substrate interfaces. Perovskite films and interfaces grown on SrTiO_3_ exhibit numerous interesting phenomena, including high-mobility quasi-two-dimensional electron gases [5-7], magnetism [8,9], and superconductivity [10,11]. Also, SrTiO_3_ is a promising candidate for the study of the growth of nanostructures. Nanostructures grown on SrTiO_3_ substrates have potential applications in the development of arrays of nanowires and quantum dots [12].

Despite the high crystalline quality of such perovskite films grown on SrTiO_3_, microscopic observations of the nucleation of perovskite films during growth have not advanced significantly due to the structural complexity and energetic instabilities arising from the volatility of oxygen [13,14]. Additionally, surface termination and reconstruction (RCs) are highly dependent on the oxygen partial pressure (*P*_O2_), as well as the temperature, which complicates any investigation of the mechanisms of film growth [15-17].

Scanning tunneling microscopy (STM) in the *diffusion-limited growth* regime allows the observation of the diffusion of single adatoms, and it has revealed the governing diffusion parameters, i.e., the surface diffusion barrier energy *E*_d_ and the attempt frequency ν_0_, for a number of metal films on metal substrates [18,19]. To apply the same methodology to the growth of perovskite oxide films, SrTiO_3_ homoepitaxy provides a simple test case.

Here, we report STM studies of nucleation behavior during the growth of SrTiO_3_ films on (2 × 1) reconstructed SrTiO_3_ (001) surfaces. Using PLD growth with low coverage and growth temperatures, we realized *diffusion-limited growth* of SrTiO_3_ films. The STM data revealed that the minimum lateral size of the SrTiO_3_ nanostructures was approximately 4 × 4 *uc*^2^ and that such primary structures expand to form one-dimensional (1D) or two-dimensional (2D) islands as either the coverage or the growth temperature increased. High-resolution STM analysis provides insight into the influence of surface RC and oxygen vacancies on the nucleation stage of island growth.

## Methods

Nb (0.1%)-doped SrTiO_3_ (001) single crystals (CrysTec GmbH) were used as substrates to obtain a sufficiently large electrical conductivity for the STM analysis. To exclude the effects of surface termination on the film growth, we removed the SrO-terminated fraction of the surface by treating the substrate in NH_4_F-buffered HF solution [20] prior to placing the samples in the growth chamber. Experiments were carried out in a combined ultra-high vacuum (UHV) STM system (with a base pressure of < 2 × 10^−10^ Torr, Omicron VT-SPM) and custom-built PLD chamber (with a base pressure of < 2 × 10^−9^ Torr). Electrochemically etched tungsten tips were used for the STM measurements. Resistive heating in the UHV chamber was used to regulate the substrate temperature *T*_sub_, which was monitored during the *in situ* surface preparation and SrTiO_3_ deposition using an optical pyrometer with emissivity of 0.8. Thermal annealing of the substrate with *P*_O2_ = 1 × 10^−2^ Torr at *T*_sub_ = 900°C was carried out for 30 min to provide an atomically flat surface with a well-ordered (2 × 1) RC and one-unit-cell (*uc*)-high (0.3905 nm) steps, as determined using reflection high-energy electron diffraction (RHEED) and STM. Growth was initiated on such TiO_2_-terminated surfaces [21-23] by ablating SrTiO_3_ single crystal targets with *P*_O2_ = 1 × 10^−4^ Torr using a KrF excimer laser (λ = 248 nm) with energy density of approximately 3 J/cm^2^ at the target surface.

## Results and discussion

**Figure 1:**
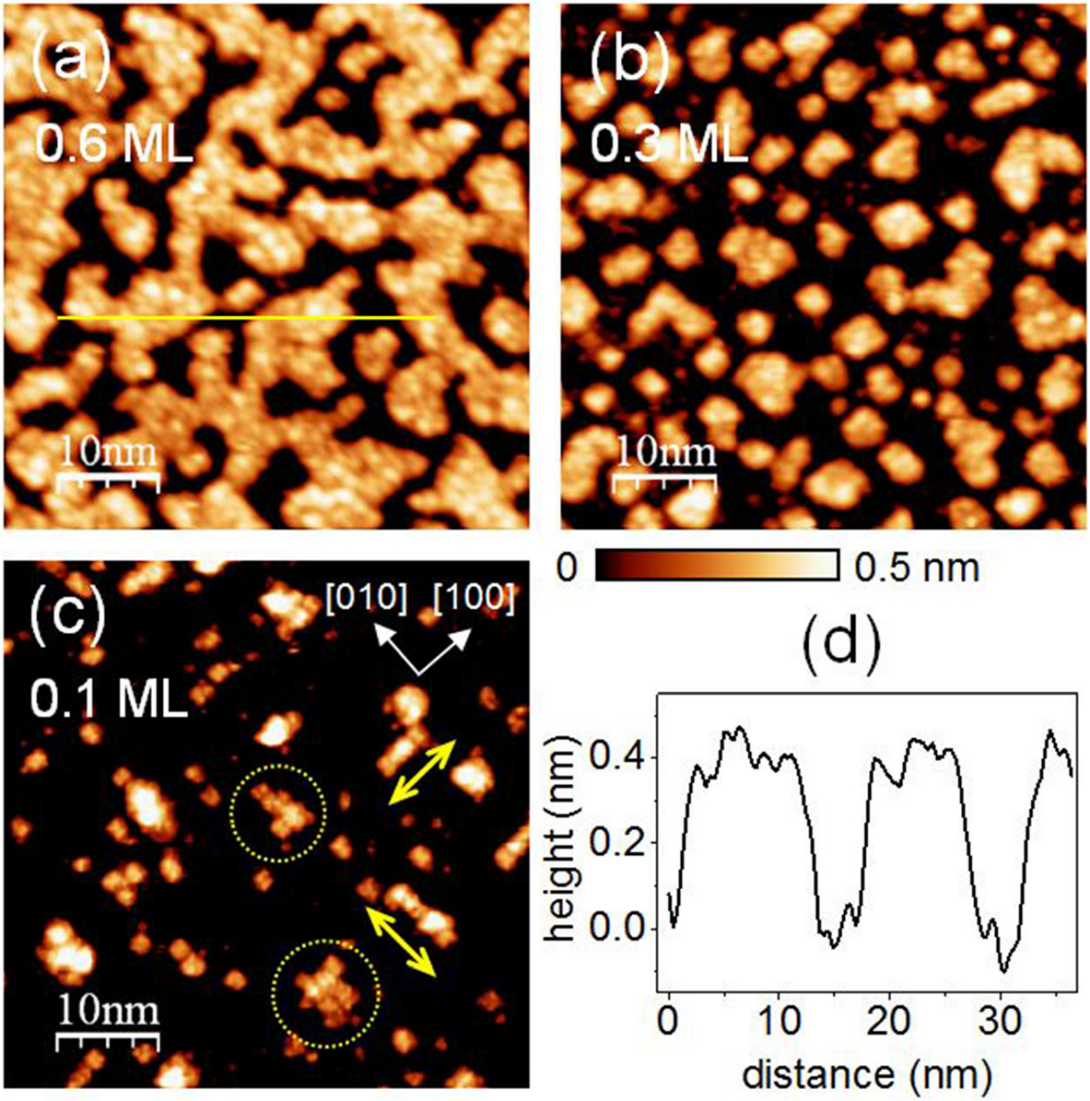
**STM images for a coverage dependence of SrTiO**_**3**_ **homoepitaxy.** The 50 × 50-nm^2^ STM images of **(a)** 0.6-ML, **(b)** 0.3-ML, and **(c)** 0.1-ML SrTiO_3_ films grown on (2 × 1) reconstructed 0.1% Nb-doped SrTiO_3_ (001) surfaces at *T*_sub_ = 580°C. The crystallographic axes are shown by the yellow arrows in (c), and some larger islands are indicated by the dotted yellow circles. All STM images were obtained with *V*_S_ = 2.5 V and *I*_set_ = 50 pA. **(d)** The height profile along the yellow line in (a).

Figure 1 shows STM topographic images for SrTiO_3_ films that were 0.6-, 0.3-, and 0.1-monolayer (ML) thick grown at *T*_sub_ = 580°C. The height profile across the surface reveals that the deposited layer is 1 *uc* high (i.e., approximately 0.4 nm), as shown in Figure 1d. When the surface coverage was greater than that of a critical coverage of approximately 0.5 ML, coalescence of islands occurred, and the film exhibited relatively homogeneous 2D growth patterns, as shown in Figure 1a, which were maintained until the coverage reached approximately 1 ML (not shown). However, the growth pattern was inhomogeneous in terms of both size and shape when the coverage was less than that required for coalescence (i.e., <0.5 ML), as can be seen for the 0.3-ML film shown in Figure 1b. The STM image of the 0.1-ML sample shown in Figure 1c reveals islands with a spatial extent of a few nanometers, which were less homogeneous with greater anisotropy than those in the 0.3-ML film. Some islands were elongated along the <100 > crystal axes of SrTiO_3_. Larger islands appear to be produced by 2D aggregation of smaller square formations.

The observations in Figure 1 draw our attention to the use of high-resolution STM in homoepitaxy of SrTiO_3_ in the *diffusion-limited growth* regime to obtain insight into the fundamental behavior of nucleation of the perovskite oxide islands. We carried out SrTiO_3_ homoepitaxy with submonolayer coverage in a *diffusion-limited growth* regime to obtain small thermally stable SrTiO_3_ islands. We inhibited island coalescence following nucleation by carrying out deposition at temperatures in the range 580 < *T*_sub_ < 620°C to reduce thermal diffusion as low as possible [17,24,25] and maximized the island-island separation by forming films using a very low coverage during the PLD process. A large laser pulse interval of 1 min was used to allow sufficient time for the deposited species to form thermally equilibrated configurations following each laser ablation event. We confirmed that the samples were fully thermally relaxed by monitoring the recovery of the RHEED intensities following each laser pulse. We prepared three sets of samples, which were distinguished by the number of laser pulses such that each set was composed of two samples grown at *T*_sub_ = 580°C and 620°C.

**Figure 2:**
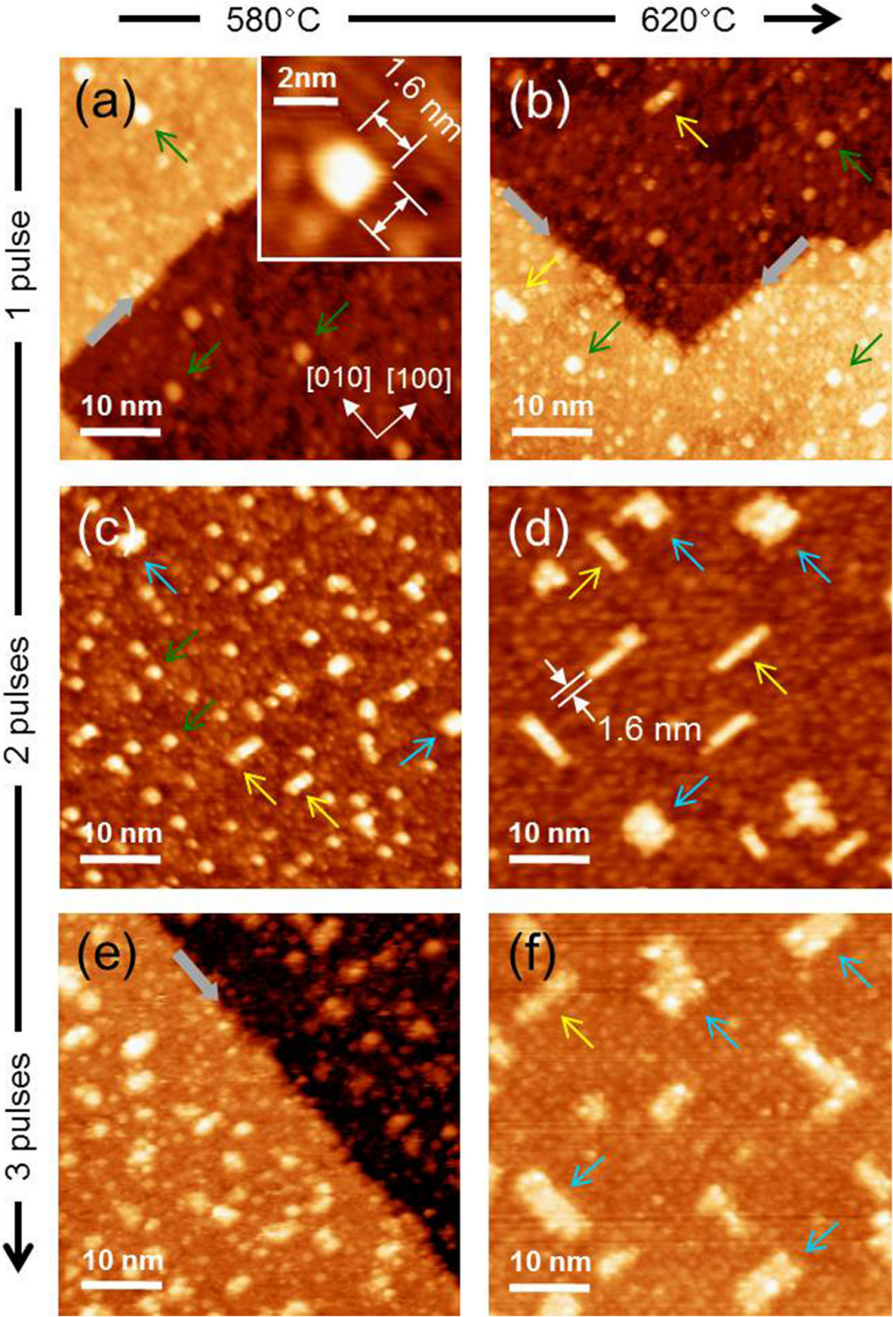
**STM images for initial growth patterns in SrTiO**_**3**_ **homoepitaxy. (a)** and **(b)** 50 × 50-nm^2^ STM images of 1-p samples grown at 580°C and 620°C, respectively. **(c)** and **(d)** (**(e)** and **(f)**) 2-p (3-p) samples grown at 580°C and 620°C, respectively. All STM images were obtained with *V*_S_ = 2.0 V and *I*_set_ = 50 pA. The green arrows indicate the smallest islands, which are 1 *uc* high. The yellow (cyan) arrows indicate 1D (2D) SrTiO_3_ islands. The inset of **(a)** shows a 7 × 7-nm^2^ STM image of a SrTiO_3_ island, as indicated by the green arrow. The gray arrows in (a), (b), and (e) indicate the substrate monatomic-high step edges.

Figure 2a and 2b show images of SrTiO_3_ films following a single pulse (1-p) at *T*_sub_ = 580°C and 620°C, respectively. The images reveal the formation of SrTiO_3_ islands of extremely small size, with a height of 1 *uc* (≈0.39 nm). At *T*_sub_ = 580°C, most of the islands were square and uniform in size. The inset of Figure 2a shows a high-resolution STM image of the smallest SrTiO_3_ island observed in this study. All four sides of the island were parallel to the directions of the crystallographic base vectors, i.e., [100] and [010]. The length of each side was approximately 1.6 nm, corresponding to 4 *uc*; hence, we term such square islands 4 × 4-*uc*^2^ SrTiO_3_. We observed larger islands that were rod-shaped at *T*_sub_ = 620°C, as shown in Figure 2b. The longer side was several times the length of the shorter side and was parallel to the crystallographic axes; interestingly, the shorter side was 4-*uc*-long SrTiO_3_.

Figure 2c shows an STM image of a 2-p sample grown at *T*_sub_ = 580°C, which exhibited both 1D and 2D islands, whereas for the 1-p sample grown at the same temperature, the dominant species was 4 × 4-*uc*^2^ SrTiO_3_ (see Figure 2a). Figure 2d shows an STM image of a 2-p sample grown at *T*_sub_ = 620°C, which exhibited very few 4 × 4-*uc*^2^ SrTiO_3_ islands and typically either 1D or 2D islands were formed. Figure 2e shows an STM image of a 3-p sample grown at *T*_sub_ = 580°C, and Figure 2f shows an STM image of 2-p sample grown at *T*_sub_ = 620°C. A similar trend can be observed, with the long axes of the 1D islands oriented along either [100] or [010], in roughly equal proportions, as may be expected from the crystal symmetry. This anisotropic growth indicates a strong anisotropy in the diffusion energy barrier of the substrate, which can be clearly distinguished from typical isotropic diffusion of metals on metal substrates [18,19].

**Figure 3:**
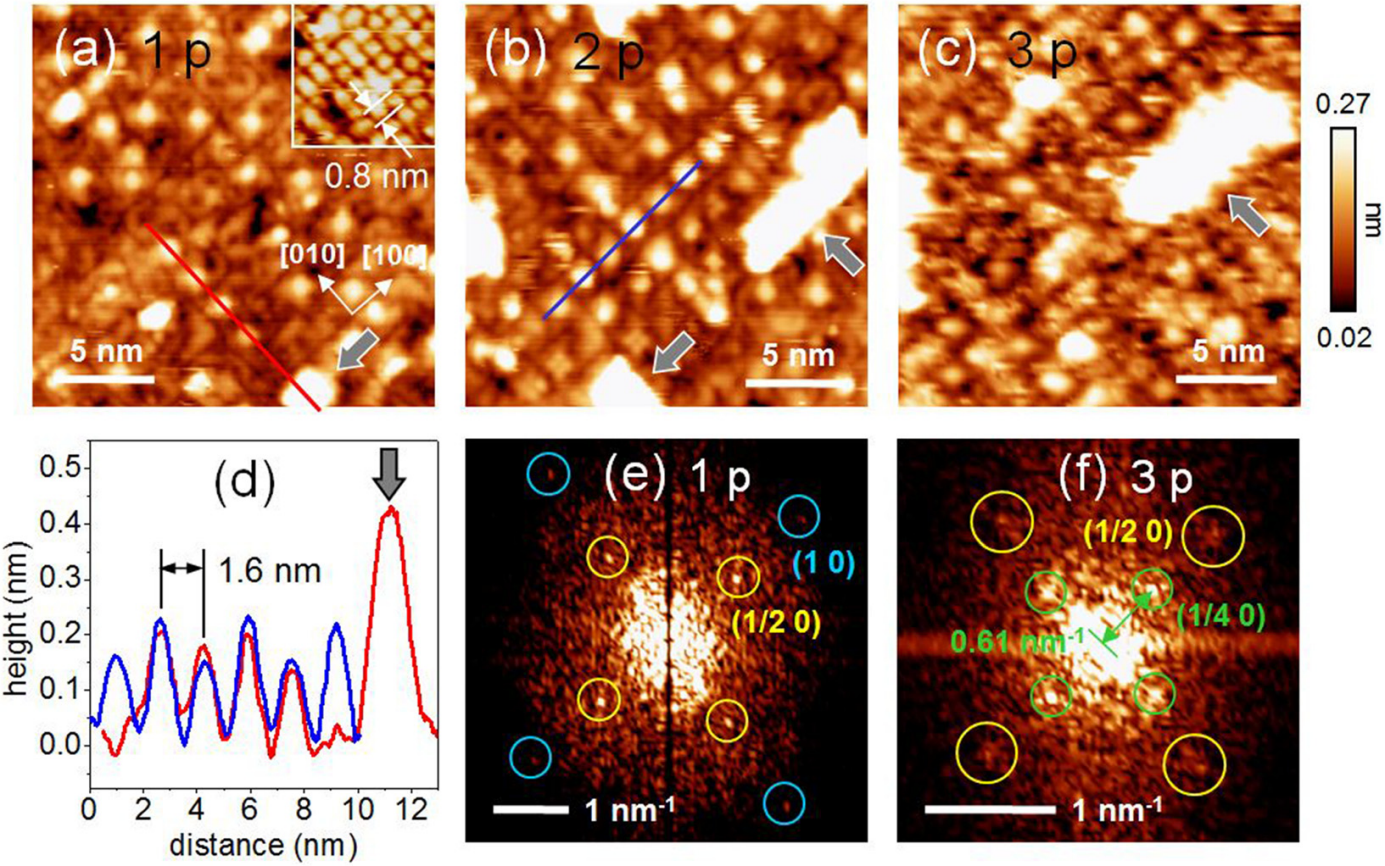
**STM images for defect formations in the initial growth stage. (a)** to **(c)** 20 × 20-nm^2^ STM images showing the formation of defects in the substrate surfaces of 1-, 2-, and 3-p samples, respectively. All STM images were obtained with *V*_S_ = 2.0 V and *I*_set_ = 50 pA. The inset of (a) shows an STM image of the same area with the same length scale that was obtained using a different tip. The gray arrows indicate large white structures, which are 1-*uc*-high SrTiO_3_ islands. The red (blue) curve in **(d)** shows the STM apparent height profile along the red (blue) line in (a) ((b)). **(e)** FT power spectrum map of (a). The yellow circles show bright spots in the FT power spectrum at (1/2 0), which are indicative of 2 × 2 RC. **(f)** FT power spectrum map of (c). The bright spots indicated by the green circles at (1/4 0) correspond to a periodicity of 4 *uc* along the directions of crystal symmetry.

The STM images shown in Figure 2 also reveal that the changes in the island growth patterns were related to the substrate surface structures. Figure 3a shows STM images of 1-p sample grown at *T*_sub_ = 620°C. We can see a 2D connection between the bright contrast of the two aligned along the two equivalent surface crystallographic directions. Similar structures have been reported for the surface of a 10-*uc-*thick SrTiO_3_ homoepitaxial film [25]. An STM image obtained with different tip conditions is shown overlaid in the inset of Figure 3a, for the same area with the same length scale, which reveals 2 × 2 periodicity. The apparent difference in the patterns of the STM contrasts measured on the same sample surface with an identical periodicity (i.e., 2 × 2) may be attributed to different contributions of the electronic structures of the tips. This reflects the importance of the local electronic structure of the tip and sample in the STM contrast rather than the sample geometry and emphasizes that care is required in the interpretation of the apparent height in STM images. To identify the symmetry of the local surface electronic structure, we carried out Fourier transform (FT) power spectrum analysis of the image in Figure 3a, as shown in Figure 3e. The bright spots at (1/2 0) in the FT power spectrum are indicative of the existence of a (2 × 2) RC [26] in the substrate surface. Theoretical modeling [14,27,28] and transmission electron microscopy [29] of the SrTiO_3_ surface suggest that oxygen vacancies form linear clusters in SrTiO_3_, which may explain the (2 × 2) RC as pairs of oxygen vacancy clusters.

The bright spots shown in Figure 3a distributed randomly in the 1-p sample; however, similar features were more ordered in the 2-p sample, as shown in Figure 3b. Such order was very rarely observed in the as-prepared substrate surface and the 1-p sample grown at *T*_sub_ = 580°C (see Figure 2a). The apparent height of these spots as determined from the STM data was in the range 0.15 to 0.2 nm, as shown in the profiles in Figure 3d. Theoretical modeling of the SrTiO_3_ surface [28] has shown that oxygen vacancies at the SrO layer exhibit high electron density. In our case, we expect that the O vacancy of the subsurface SrO layer may induce a similar change in the local electron density of the TiO_2_ layer at next above the vacancy position, which might contribute to the enhanced STM contrast of the apparent defects on the surface. Interestingly, we observed that these defects were aligned along the crystal symmetry directions. The separation of these defects was 1.6 nm (approximately 4 *uc*), as identified from the line profiles shown in Figure 3d. Moreover, a 2D expansion of an array of these defects was observed as the defect density increased, as shown in Figure 3b, c. In the 3-p sample shown in Figure 3c, such arrays of defects fully occupied the surface with 2D 4 × 4-*uc* periodicity. This periodicity is also apparent in the FT power spectrum map of the image in Figure 3c, as shown in Figure 3f, where 4-*uc* periodicity can be observed along the crystal symmetry directions.

Stable configurations of the primary SrTiO_3_ islands occurred in parallel with the development of the defect-induced RC formations, and the geometry of these features was related to the crystallographic symmetry of the SrTiO_3_ (001) surface. Phark et al. reported [17] the failure of expitaxial growth, together with the absence of notable changes in the surface structure during deposition, for homoepitaxy on a (6 × 2) RC surface. This supports the possibility of defect-induced surface RC formations, i.e., 4 × 4-*uc* periodic arrays of defects, as a prerequisite or precursor that is significant in the activation of nucleation and growth during SrTiO_3_ homoepitaxy. Further theoretical work may expand our understanding of the connection between the surface RC and the initial stages of growth during SrTiO_3_ homoepitaxy.

Careful attention was paid to the growth conditions in this study, which enabled us to observe the nucleation of SrTiO_3_ islands and the growth of perovskite nanostructures via the addition of SrTiO_3_ molecular units. This work makes an important contribution to conventional diffusion/nucleation theory for metal-on-metal epitaxy, extending it to complex oxide systems. Further investigation should be carried out to characterize the minimum nucleation size and the energy *E*_d_ to improve our understanding of the differences in the diffusion characteristics of the perovskite-perovskite systems compared to metal-metal epitaxy.

## Conclusions

We have investigated the growth of SrTiO_3_ islands on SrTiO_3_ (001) surface using PLD and *in situ* STM. We found that 4 × 4-*uc*^2^ islands form as nucleation seeds, which grow along the crystal axes with a width of 4 *uc*. This result suggests that energetically stable nucleation of SrTiO_3_ islands is related to (2 × 2) surface reconstruction, with the formation of 4-*uc* periodic arrays of defects. Our observations represent an atomic-scale approach to understand the initial stages of growth of SrTiO_3_ and the importance of the energetic stability of SrTiO_3_ (001) surface RC via oxygen vacancies in the perovskite crystal.

## Abbreviations

FT: fourier transform
ML: monolayer
PLD: pulsed laser deposition
RC: reconstruction
RHEED: reflected high energy electron diffraction
STM: scanning tunneling microscopy
UHV: ultra-high vacuum
uc: unit-cell
